# Inter-relationships between perception of educational environment and learning processes within medical undergraduate psychiatry teaching: a mediational analysis

**DOI:** 10.1080/10872981.2021.1998944

**Published:** 2021-10-31

**Authors:** Qian Hui Chew, Eelin Tan, Min Yi Sum, Kang Sim

**Affiliations:** a Research Division, Institute of Mental Health, Singapore; b Radiology, Kk Women’s and Children’s Hospital, Singapore; c West Region, Institute of Mental Health, Singapore

**Keywords:** Educational environment, motivation to learn, engagement, learning outcomes, medical education, psychiatry

## Abstract

Amongst medical undergraduates, the perception of educational environment (EE) has been associated with academic achievement and positive attitude toward the course. Nonetheless, there are sparse data on how it influences various learning processes and outcomes especially within psychiatry training. Consistent with situativity and self-determination learning theories, we hypothesized that a positive perception of the EE within psychiatry postings will be beneficial for the learning process, specifically pertaining to greater motivation to learn, better engagement, allowing them to feel more equipped, and greater appreciation of the subject. The DREEM (Dundee Ready Education Environment Measure) was administered to fourth-year medical undergraduate students from the Yong Loo Lin School of Medicine, Singapore, undergoing psychiatry rotations from 2015 to 2019. The students also completed five additional items evaluating the specific learning processes (motivation to learn, engagement, equipping, and appreciation of the subject) and overall rating of the posting. We examined the relationship between DREEM domains and learning processes using correlation analysis. We explored learning processes as mediators of the relationship between total DREEM scores and overall rating of the posting. Altogether, 1343 (response rate 89.5%) medical undergraduates participated in the study. The overall DREEM score was 157.01 ± 15.86. Overall DREEM and subdomain scores were significantly correlated with several learning processes (r = 0.354 to 0.558, all p < .001). Motivation and engagement were significant mediators of the relationship between total DREEM scores and overall rating of the psychiatry posting. Our results highlighted that a positive perception of EE was associated with the specific learning processes that mediated the overall rating of the posting. In the context of relevant learning theories and our study findings, improvement of the EE within undergraduate psychiatry training can potentially enhance overall learning experience through better motivation and engagement of our learners.

## Introduction

The perception of the educational environment (EE) by learners reflects their learning experience [1], and a negative perception could adversely affect cognitive and affective outcomes, academic achievement, and attitudes [2–5]. Various definitions of the educational environment have been proposed, and can be defined as ‘a dynamic, complex structure with multiple inter-related and interactive facets that involve the trainee, the trainee’s interactions with his peers, supervisors, and other members of the team, the training program and the structure of the organization that one works in’ [6]. Understanding the learner’s perception of their EE allows better evaluation of the different learning contexts [4,7] and attention towards specific areas in order to enhance and optimise the EE in terms of teaching, role autonomy and support [8–10]. Previous studies have evaluated the impact of perception of EE on outcomes such as quality of life [11,12], academic performance [13], and career choice [14]. There are several widely used measures capable of reliably capturing this construct. One such tool is the Dundee Ready Education Environment Measure (DREEM), which has been extensively used in the evaluation of EE within undergraduate medical education [1,15]. To date, there have been few studies that examine the relationship between perception of the EE and specific learning processes, especially in psychiatry postings amongst our medical undergraduates [15]. Based on a recent review of the DREEM and its use in undergraduate learning environments, only one study explored its relationship with approaches to learning [16]. A better understanding of the inter-relationships between learners’ perception of the teaching, role autonomy, and social support with learning processes and outcome can help to identify aspects of EE that can be enhanced to improve the learning experience during psychiatry postings within our medical undergraduates.

### Theories of learning

Situated cognition and situated learning theories propose that thinking and learning, respectively, are situated in experience [17]. Learning activities should hence be conducted in the authentic environment and culture of the discipline being studied [18], which optimizes the context for learning [19]. Learning will result from the student’s interaction with their environment [17]. The learning process comprises several essential components derived from learning theories such as self-determination and constructivist theories, among others [20–26]. This includes motivation, engagement, appreciation, and equipping [21,23,26]. Motivation explains why students learn [20], and intrinsic motivation has been thought of as the desired type of motivation [21]. For one to be intrinsically motivated, desires for autonomy, competence, and relatedness must be fulfilled, as described in Deci and Ryan’s [22] theory of self-determination. Engagement represents how psychologically and cognitively invested students are in learning [23], and can be thought of as the degree of purposeful involvement with learning activities [24]. Engaged students are attracted to their work, persist despite challenges, and take pride in accomplishment [25]. Motivation and engagement can be inter-related [23] in that the more motivated one is, the more likely he or she is to be engaged. In the learning process, students also construct meaning through learning experiences that prompt reflection and internal processing which can lead to better equipping and appreciation of the subject matter [26]. Being successfully equipped in the subject matter means that the learner has been able to transform the knowledge gained into a format that allows them to present this material to others, internalize and reflect upon it, and use it to answer questions [27,28]. Despite recent efforts to explore perception of the learning environment and its correlations to specific learning processes [29], it still remains unclear if positive perception of learning environment alone leads to overall satisfaction with the course, or whether it is the increased motivation and appreciation of the subject matter gained through independent learning in a conducive environment that determines learners’ satisfaction.

### Aims of study

In this cross-sectional study, we had two aims. First, we sought to evaluate the perception of the EE of undergraduate medical students following their completion of their psychiatry rotations at the Institute of Mental Health (IMH), the only tertiary psychiatric hospital in Singapore.

Second, we sought to determine if perception of EE relates to the different learning processes, namely (i) motivation to learn about psychiatric disorders, (ii) engagement with the discipline of psychiatry, (iii) appreciation of relevant topics in psychiatry, and (iv) how equipped students feel about their understanding of psychiatric conditions.

Based on extant data, we hypothesized that a positive perception of the EE would be associated with specific learning processes in our cohort of medical undergraduates undergoing their psychiatry rotations which mediated the overall rating of the posting.

## Materials and methods

### Participants

The study was conducted from 2015 to 2019. Batches of fourth-year undergraduate students from the Yong Loo Lin School of Medicine (YLLSOM) at the National University of Singapore undergo a psychiatry rotation lasting 6 weeks that comprises generally of a week of academic teaching at the University, 2 weeks attachment at a tertiary psychiatric hospital and 3 weeks attachment within a department of psychological medicine at a general hospital setting. Those undergraduate students who completed a two-week Psychiatry rotation at IMH were administered the DREEM questionnaire that surveyed their perception of various facets of the EE in relation to this particular rotation. The only demographic information collected was the gender of the student. All procedures performed in this study were in accordance with the Declaration of Helsinki. The study was approved as an exempt study by the Institutional Review Board of the National Healthcare Group, Singapore (NHG DSRB Ref: 2014/00422).

### Measures

The DREEM questionnaire consists of 50 items rated on a 5-point Likert scale ranging from 0 (strongly disagree) to 4 (strongly agree). Negatively worded items were reverse coded, and total scores could be obtained for five domains: i) Student Perception of Learning (SPL; Maximum score 48), ii) Student Perception of Teachers (SPT; Maximum score 44), iii) Student Perception of the Atmosphere of the Learning Environment (SPA; Maximum score 48), iv) Student Academic Self-Perceptions (SASP; Maximum score 32), and v) Student Social Self-Perceptions (SSSP; Maximum score 28) [30,31]. Scores were interpreted based on the cut-offs given in the DREEM guide [31]. The sum of all items was also calculated for a total DREEM score. The maximum cumulative score is 200, whereby higher scores indicate a more positive perception of the EE [32]. This questionnaire has previously been administered to psychiatry undergraduate students in Singapore [14], and found to be consistently reliable regardless of countries, cultures, and settings [15].

In addition to the 50 standard DREEM items, our questionnaire included five items that served to measure various aspects of learning. The five items specifically explored (i) one’s motivation to learn about psychiatric disorders, (ii) level of engagement with the topic of psychiatry, (iii) one’s appreciation of the topic, (iv) how equipped one felt about managing psychiatric conditions, and (v) one’s overall rating of the rotation. The items above were rated on a five-point Likert scale ranging from Strongly Disagree to Strongly Agree. The item on Overall Rating of Session was rated on a five-point Likert scale corresponding to a continuum of Poor, Below Average, Average, Above average, and Excellent. The course instructors were not personally involved in the collection of the forms in order to reduce the risk of coercion. Instead, participants were instructed to leave their completed anonymous forms in a collection box prior to leaving the class.

### Data analysis

All analyses were conducted using IBM SPSS 23 (IBM Corp, Armonk, NY). We first carried out descriptive analyses of DREEM overall and subdomain scores and examined DREEM score differences between genders using independent t-tests. Pearson correlations of the DREEM subdomains with the four learning processes and overall rating of posting were then performed. Statistical significance was set a priori at an alpha of 0.05 (two-tailed). Finally, we tested a mediation model using the PROCESS macro [33] to explore if the relationship between total DREEM score and overall rating of the posting was mediated by specific learning processes. The DREEM scores, scores on learning processes, and overall rating of the posting were standardized before we entered it into a mediation model in order to obtain standardized coefficients.

## Results

### Descriptive statistics

We obtained data from 1343 students who participated in the study (Females 716, 53.3%) out of 1500 students, with a response rate of 89.5%. Overall DREEM score was found to be in the highly positive range [31] (Table 1). SPL, SPT, and SPA domain scores were in the top quartile, suggesting that students thought positively of the teaching, of their teachers, as well as the atmosphere of the EE [31]. The remaining two domains were in the upper third quartile [31]. The median scores of items about learning and the overall rating of the posting are displayed in Table 2.

**Table 1.** Scores for Dundee Ready Education Environment Measure (DREEM) domains

DREEM domain	Mean (S.D)/Maximum score	Percentage score
SPL	37.68 (4.29)/48	78.5
SPT	37.04 (3.90)/44	84.2
SASP	23.41 (3.15)/32	73.2
SPA	37.75 (4.43)/48	78.6
SSSP	21.08 (2.83)/28	75.3
Total	157.01 (15.86)/200	78.5

Abbreviations: SPL = Student Perception of Learning; SPT = Student Perception of Teachers; SASP = Student Academic Self-Perception; SPA = Student Perception of the Atmosphere of the Learning Environment; SSSP = Student Social Self-Perception.

**Table 2.** Mean scores for learning domains and overall rating of posting

		Median	IQR
Items about learning	I am more motivated to learn psychiatric disorders	3	1
	I am more engaged in psychiatry	3	1
	I better appreciate relevant psychiatric topics	3	1
	I am better equipped to manage psychiatric conditions	3	1
Overall rating	Overall rating of the posting	3	1

Note: Maximum score is 4.

Abbreviation: IQR = Interquartile range.

Our results also showed that there were statistically significant correlations of the cumulative DREEM score and all DREEM domains with items measuring impact on learning processes and overall rating of the posting (see Table 3).

**Table 3.** Pearson correlations among Dundee Ready Education Environment Measure (DREEM) scores, learning processes, and rating of posting

	SPL	SPT	SASP	SPA	SSSP	DREEM
More motivated to learn psychiatric disorders	0.462**	0.377**	0.391**	0.473**	0.419**	0.503**
More engaged with psychiatry	0.470**	0.389**	0.410**	0.489**	0.408**	0.514**
Better appreciation of relevant psychiatric topics	0.433**	0.375**	0.354**	0.450**	0.367**	0.470**
Better equipped at management psychiatric conditions	0.468**	0.360**	0.437**	0.494**	0.388**	0.509**
Overall rating of posting	0.541**	0.455**	0.370**	0.519**	0.456**	0.558**

Note: **Correlation is significant at the 0.01 level (2-tailed).

Abbreviations: SPL = Student Perception of Learning; SPT = Student Perception of Teachers; SASP = Student Academic Self-Perception; SPA = Student Perception of the Atmosphere of the Learning Environment; SSSP = Student Social Self-Perception.

There was a significant difference between genders regarding SASP (t(1337) = 2.541, p = .011), with males having a better academic self-perception (M = 23.64, S.D = 3.21) than females (M = 23.20, S.D = 3.09). Males also gave higher scores than females on overall rating of the posting (t(1338) = 2.18, p = .030) (M = 3.46, S.D = 0.57 for males vs. M = 3.39, S.D = 0.60 for females).

### Mediation

**Figure 1.**
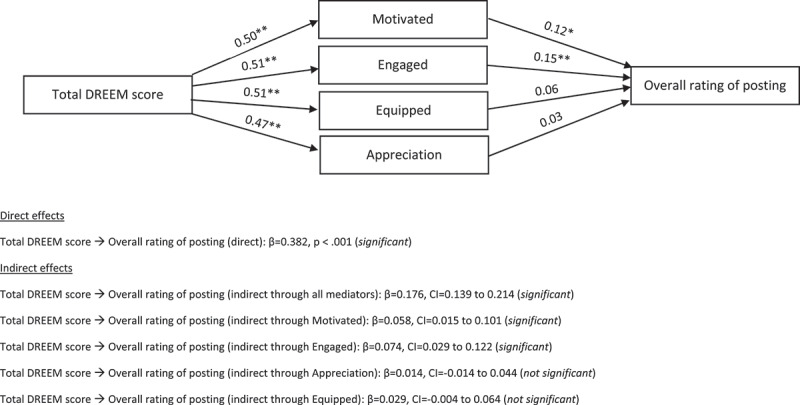
Effect of total DREEM score on overall rating of posting with learning processes as parallel mediators

Notes: All indirect effects are reported below the figure, and are completely standardized. *p < .05, ** p < .001.

Fourteen participants who had missing data on one or more variables to be included in the mediation model were excluded from the analysis. There was a direct effect of total DREEM scores on overall rating of the posting. There was also an indirect effect of total DREEM score on overall rating of posting, with the learning processes of motivation and engagement being two significant mediators in the model. On the other hand, appreciation of the topic and feeling more equipped were not significant mediators. The results are presented in Figure 1.

## Discussion

There were several main findings from this study. First, amid positive overall DREEM and subscale scores, there were significant gender differences (with males indicating a more positive rating) regarding academic self-perception and overall rating of the posting. Second, there were statistically significant correlations of the DREEM overall and subscale scores with specific learning processes and overall rating of the posting. Third, the learning processes of motivation and engagement were significant mediators of the relationship between total DREEM scores and overall rating of the posting.

The overall DREEM scores in this study were higher compared with several earlier studies in Asia [34–37] but comparable with similar studies in Australia [38] and Ireland [39,40]. Our overall DREEM score was consistent with the results obtained in a previous Singapore-based study on undergraduate students undergoing a psychiatry rotation although at a different site [14]. Our study noted that male students tended to have better academic self-perception, and better overall rating of the posting which add to the heterogeneous literature findings. For example, while several studies have shown no difference in DREEM scores between genders [38,41–43], others reported that female students perceived EE more positively than males [36,44], and the remaining studies have reported the converse [30,45,46].

**Figure 2.**
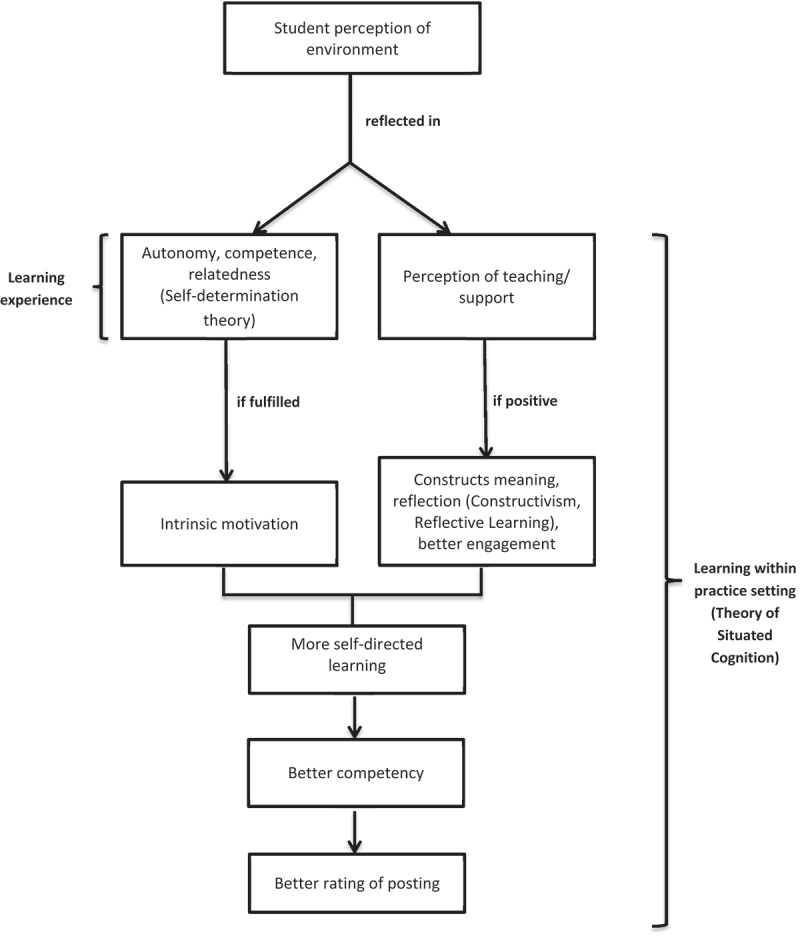
Relationship between perception of learning environment, specific learning processes and overall rating of posting

We found correlations between DREEM scores and learning processes highlighting inter-related and interactive features between these components. In addition, our mediation model sought to explore if the relationship between the EE and students’ overall rating of the posting was indeed mediated by the specific processes of learning. The components of motivation and engagement were the two significant mediators, while appreciation of the topic and feeling more equipped were not. Figure 2 integrates our proposed relationship between perception of EE (overall and subdomains) and specific learning process mediators (motivation, engagement) with the overall rating of the posting. Relevant learning theories have been incorporated into the specific components to highlight the inter-related aspects of the different variables and related learning theories. For instance, in attending to patients with psychiatric conditions within the clinical setting (theory of situated learning [17]) which was deemed to encourage role autonomy and with supportive teachers (self-determination theory [22]), the students could be better motivated, more engaged, and reflective (constructivism, reflective learning) of the conditions experienced by these patients. This could lead to better self-directed learning about the different psychiatric conditions, management modalities and prognosis, thus increasing their competency and deepening their understanding. This in turn positively influences their overall impression of their learning within the posting. Future studies may seek to validate this model with longitudinal follow-up and qualitative interviews of the learners.

The study findings outline several practical implications for training programs. First, it highlights the importance of knowing and continually improving the EE together with the efforts of the teacher and learner within the undergraduate learning journey in psychiatry. Second, the overall DREEM and specific subscale scores could serve as program feedback on the areas of EE that warrant further attention or continual efforts including areas of teaching, learning, encouragement of role autonomy, and garnering of social support for the learners. Third, there is high correlation between aspects of EE and specific learning processes. Understanding this encourages adequate and continual attention being paid to the development of a conducive EE for our undergraduate learners that can foster greater motivation and engagement. Consistent with adult learning theory [47,48], better motivation facilitates reflection and self-directed learning that can positively impact on the learning of psychiatry.

There are several limitations. First, the items were self-reported and may be affected by other factors apart from the EE such as personal life events and personality factors. Second, this study is cross-sectional in nature and is limited in its ability to provide deeper insights into the subjective mechanisms behind the findings reported. Future studies may want to capture additional qualitative comments to supplement the quantitative data to allow a richer appreciation of the details regarding specific domains in the EE. Longitudinal follow-up of the students and determination of how their appreciation of EE impacts on overall progress and assessment outcomes at the end of the year could be considered. Third, taking into consideration possible effects of respondent fatigue, the four learning processes and overall rating of the posting were assessed using single-item measures. This may not have adequately captured all facets of motivation, appreciation, engagement, and equipment of the subject matter. In addition, the items were all positively framed, which may increase the risk of response bias. Future studies may seek to expand on the items to further assess learning processes.

In conclusion, we found that the perception of the EE had a significant impact on overall rating of posting which were mediated through learning processes such as motivation and engagement. In the context of relevant learning theories and our study findings, improvement of the EE within undergraduate psychiatry training can potentially enhance overall learning experience through better motivation and engagement of our learners.

## Data Availability

The datasets used and/or analysed during the current study are available from the corresponding author on reasonable request.
